# Population-based type-specific prevalence of high-risk human papillomavirus infection in Estonia

**DOI:** 10.1186/1471-2334-10-63

**Published:** 2010-03-11

**Authors:** Anneli Uusküla, Mart Kals, Liina Kosenkranius, Louise-Anne McNutt, Jack DeHovitz J

**Affiliations:** 1 Department of Public Health, University of Tartu, Tartu, Estonia; 2 Quattromed HTI Laboratories Ltd, Tartu, Estonia; 3 Department of Epidemiology, School of Public Health, State University of New York, Albany, USA; 4 Department of Preventive Medicine and Community Health, State University of New York Downstate Medical Center, Brooklyn, USA

## Abstract

**Background:**

Effective prophylactic vaccines are available against human papillomavirus (HPV) types 6, 11, 16, and 18 which are licensed for routine use among young women. Monitoring is needed to demonstrate protection against cervical cancer, to verify duration of protection, and assess replacement frequency of non-vaccine types among vaccinated cohorts.

**Methods:**

Data from a population-based study were used to assess the type-specific prevalence of HPV in a non-vaccinated population in Estonia: 845 self-administered surveys and self-collected vaginal swabs were distributed, 346 were collected by mail and tested for HPV DNA from female participants 18-35 years of age.

**Results:**

The overall HPV prevalence (weighted estimate to account for the sampling method) in the study population (unvaccinated women aged 18-35) was calculated to be 38% (95% CI 31-45%), with estimated prevalences of high- and low-risk HPV types 21% (95% CI 16-26%), and 10% (95% CI 7-14%), respectively. Of the high-risk HPV types, HPV 16 was detected most frequently (6.4%; 95% CI 4.0-9.8%) followed by HPV 53 (4.3%; 95% CI 2.3-7.2%) and HPV 66 (2.8%; 95% CI 1.3-5.2%).

**Conclusions:**

We observed a high prevalence of total and high-risk type HPV in an Eastern European country. The most common high-risk HPV types detected were HPV 16, 53, and 66.

## Background

Human papillomavirus (HPV) is thought to be the most common sexually transmitted infection in the world [1]. Genital HPV types are categorized according to their epidemiological association with cervical cancer. Infections with low-risk types, such as HPV types 6 and 11, can cause benign or low grade changes in cervical cells, genital warts, and recurrent respiratory papillomatosis. High-risk HPV types can cause cervical, anal, and other genital cancers. High-risk HPV types are detected in 99% of cervical cancers, and worldwide approximately 70% of cervical cancers are due to HPV types 16 and 18 [2]. Many developed countries have licensed vaccines that are highly effective against HPV types 6, 11, 16, and 18 for prophylactic use among young females [3]. With widespread use of the vaccine, decreases in the prevalence of HPV types covered by the vaccine would be expected [4]. Close monitoring is needed to demonstrate protection against cervical cancer, to verify duration of protection, and assess the replacement frequency of non-vaccine types among vaccinated cohorts, and potential barriers to vaccination coverage [5,6]. However, before a vaccination program is undertaken, baseline HPV disease burden must be assessed to gauge the efficacy of the program and the vaccine itself. To date, there are limited data on the prevalence of HPV among women living in eastern European countries. We have therefore studied the pre-vaccine prevalence of different types of HPV in Estonia.

## Methods

### Sample

An age-stratified (18-20, 21-25, 26-30, 31-35) random sample of 845 women aged 18-35 years was derived from the Estonian Population Registry list for Tartu city (the second largest city in the country) and county residents (from a total of 22,904 women aged 18-35). The Estonian Population Registry is an electronic database containing personal data about people residing in Estonia.

### Study procedures

Data collection was conducted from September 2005 to May 2006. Each participant was mailed a package containing a specimen collection kit (a cotton swab for vaginal swabbing on a plastic handle packed in an individual reclosable plastic sleeve; Eurotubo^® ^Collection swab, Deltalab, S.L.I., Barcelona, Spain), collection instructions and an informed consent form attached to a 35-item questionnaire requesting socio-demographic characteristics, sexual behaviour, and health care utilization data. For specimen collection, participants were instructed to wash their hands before opening the swab, to hold the swab by the end of the handle, to insert the foam swab into the vagina as if inserting a tampon, to gently turn the swab during a count of 10, and to replace the swab in the plastic sleeve, avoiding contact with the external genitalia.

Self-collected specimens (vaginal swabs) were mailed directly to the laboratory, in pre-stamped, pre-addressed envelopes. The swabs were transported in a dry state. Earlier studies have documented good performance of NAAT assays for the detection of HPV (and other genital pathogens) using self-obtained vaginal swabs shipped in a dry state to a laboratory [7-10]. Samples were tested in the ISO 15 189 accredited diagnostics laboratory of Quattromed HTI Laboratories Ltd. Study procedures complied with local regulations regarding mailing of biological materials. (Detailed description of the study design is provided elsewhere [11].)

### Laboratory methods

#### Specimen processing and DNA isolation

The vaginal swabs were held for up to three days at 4°C prior to DNA extraction. Material from swab specimens was suspended in PBS and collected by centrifugation at 16,060G for 20 minutes. The supernatant was discarded and the pellet was resolved in PBS. DNA was extracted using the High Pure PCR Template Preparation Kit (Roche Molecular Biochemicals, Mannheim, Germany) according to the manufacturer's instructions. Amplification of the human β-globin gene was performed to confirm the integrity of the DNA in the samples [12].

#### HPV genotyping

Human papillomaviruses were detected by single-round PCR using the degenerated oligo primers MY09/11 and HMB01 as described by Gravitt et al. [13] using an Eppendorf Mastercycler (Hamburg, Germany). The PCR products were detected by ethidium bromide-stained agarose gel electrophoresis. All positive results were genotyped by restriction fragment length polymorphism analysis as described in Mejer et al. [14] The HPV genotypes were classified using the systems proposed by Dunne EF et al. and Munos et al. [4,15] The following HPV types were classified as high-risk: 16, 18, 31, 33, 35, 39, 45, 51, 52, 56, 58, 59, 68, 73, 82 including probable high-risk types 26, 53, 66. Types 6, 11, 40, 42, 43, 44, 54, 61, 70, 72, 81 and 89 were classified as low-risk.

### Statistical analysis

The HPV prevalence was calculated from the number of positive cases divided by the number of tested specimens. Weighted estimates of population prevalence were computed to adjust for the stratified sampling utilized in the study. Correlates of HPV prevalence were explored using the chi-square test for proportions, and multiple logistic regression analysis to assess confounding and interaction between variables. Factors with p < 0.05 in bivariate analysis were entered in a multivariate comparison. A conceptual framework approach was used for the multivariable analysis [16]. All analyses were performed with statistical software R (version 2.2.1 for Windows).

Ethical approval was obtained from the Ethics Review Board of the University of Tartu and the Institutional Review Board of the State University of New York at Albany. The study followed data protection legislation requirements from the Estonian Data Protection Inspectorate. Study participants received no financial incentive for participation.

## Results

### Study sample and socio-demographic characteristics

Study invitations reached 86% (723/845) of the targeted sample but 122 individuals could not be reached because the packet was undeliverable (e.g., moved, wrong address). These subjects were excluded from the analysis.

Women who returned the questionnaire or self-collected a vaginal sample (n = 346) were compared with those who did not (n = 377) using population registry data. There was no statistically significance difference in response proportion according to age, residency or ethnicity (data not shown) [11].

Respondents ranged in age from 18 to 35 years (mean 26.9, SD 5.5). Two out of three participants were either married (23%) or living with a sexual partner (41%).

### HPV DNA Prevalence Overall and by Age

Of the 326 adequate specimens available for HPV DNA testing, 37% (95% CI 31-44%) were positive for any HPV DNA. After weighting for the source population distribution, the prevalence estimate was 38% (95% CI 31-45%) (Table 1). While the overall HPV prevalence was highest in the 21-25-year age group, greater high- risk HPV prevalence was documented for the youngest age group (aged 18-20 years).

**Table 1:** HPV prevalence among women in Estonia by age group (data from DNA analysis of self-collected vaginal swabs, 2006)

*Age group* Number of women	*18-20* N = 52	*21-25* N = 95	*26-30* N = 75	*31-35* N = 104	*Overall*^*a*^
					

**Any HPV**					
Prevalence (%)	44	46	31	29	38
95% CI	28-66	34-62	19-46	19-41	31-45
					
**Low-risk types**					
Prevalence (%)	12	12	11	7	10
95% CI	4-25	6-21	5-21	3-14	7-14
					
**High-risk types**					
Prevalence (%)	27	23	16	17	21
95% CI	15-45	15-35	8-28	10-27	16-26
					
**Unknown risk types**					
Prevalence (%)	6	12	4	5	7
95% CI	1-17	6-21	1-12	2-11	4-10

^*a *^Weighted estimates of population prevalence (adjusted for the stratified sampling utilized in the study design)

### Prevalence of High-Risk, Low-Risk, and Specific Types

The overall prevalence of high- and low-risk HPV types was 21% (95% CI 16-26%) and 10% (95% CI 7-14%), respectively. The prevalence of unidentified HPV types was 7% (95% CI 4-10%). The most common HPV types detected were HPV 16, 53, 66 (high-risk types), and HPV 61, 81 (low-risk types) (Table 2).

**Table 2:** Type-specific HPV DNA prevalence amongst women in Estonia, 2006

	Number	Prevalence (%)	95% CI
			

**High-risk HPV**			

HPV 16	21	6.4	4.0-9.8

HPV 53	14	4.3	2.3-7.2

HPV 66	9	2.8	1.3-5.2

HPV 31	7	2.1	0.9-4.4

HPV 51	7	2.1	0.9-4.4

HPV 58	6	1.8	0.7-4.0

HPV 33	5	1.5	0.5-3.6

HPV 18	2	0.6	0.01-2.2

HPV 45	2	0.6	0.01-2.2

HPV 39	1	0.3	0-1.7

HPV 52	1	0.3	0-1.7

HPV 82	1	0.3	0-1.7

			

**Low-risk HPV**			

HPV 61	16	4.6	2.6-7.6

HPV 81	8	2.5	1.1-4.8

HPV 83	4	1.2	0.3-3.1

HPV 54	3	0.9	0.2-2.7

HPV 62	2	0.6	0.01-2.2

HPV 72	2	0.6	0.01-2.2

HPV 84	2	0.6	0.01-2.2

HPV 6	1	0.3	0-1.7

HPV 11	1	0.3	0-1.7

			

HPV 6 or 11	2	0.6	0.01-2.2

HPV 16 or 18	23	7.1	4.5-10.6

Only one HPV type	83	25.5	20.3-31.6

Multiple HPV types	15	4.6	2.6-7.6

### Factors Associated With HPV DNA Detection

Detection of HPV DNA was significantly correlated in the bivariate analysis with age, and sexual behaviour (ever having sex, young age at debut, and higher number of sexual partners within the last 12 months). However, HPV DNA was detected in 6% of females who reported never having had sex. The final multivariate model demonstrated that age (> 26, OR = 0.4, 95% CI 0.2-0.9), age above 18 years at first sexual intercourse (OR = 0.4, 95% CI 0.2-0.8), and increasing numbers of sexual partners in the last 12 months (OR = 2.5, 95% CI 1.4-4.5) were independently associated with HPV DNA detection (Table 3).

**Table 3:** Bivariable and multivariable factors associated with HPV DNA positivity amongst women in Estonia, 2006

			Bivariate analysis			Multivariable analysis		
	HPV +/Total	%	OR	95% CI	p-value	OR	95% CI	p-value
**Characteristic**								
**Socio-demographic**								
*Age (years)*								
< = 20	23/52	44.2	1			1		
21-25	44/95	46.3	1.1	0.6-2.1		0.8	0.4-1.7	
26-30	23/75	30.7	0.6	0.3-1.2		0.4	0.2-1.0	
30+	30/104	28.8	0.5	0.3-1.0	0.03	0.4	0.2-0.9	0.05
								
*Ethnicity*								
Estonian	96/269	35.7	1					
Other	22/52	42.3	1.3	0.7-2.4	0.4			
								
*Marital status*								
Married	23/81	28.4	1					
Co-habiting	56/143	39.2	1.6	0.9-2.9				
Never married	31/81	38.3	2.2	0.8-6.5				
Other	8/17	47.1	1.6	0.8-3.0	0.3			
								
*Education (in years)*								
< = 9 years	8/19	42.1	1					
10-12 years	32/88	36.4	0.8	0.3-2.2				
13+ years	72/202	35.6	0.8	0.3-2.0	0.9			
								
**Sexual behavior**								
*Ever had sexual intercourse*								
Yes	117/306	38.2	1					
No	1/16	6.3	0.1	0.01-0.8	0.03			
								
*Age at the first intercourse*								
< = 18 y	95/217	43.8	1			1		
> 18 y	20/85	23.5	0.4	0.2-0.7	0.001	0.4	0.2-0.8	<0.01
								
*Number of sexual partners in last 12 months*								
1	72/226	31.9	1			1		
2-5	39/67	58.2	3.0	1.7-5.2	< 0.001	2.5	1.4-4.5	< 0.01
> = 6								
								
*Self-reported history of sexually transmitted infections*								
Yes	33/82	40.2	1					
No	83/233	35.6	0.8	0.5-1.4	0.5			
								
**Health behaviour**								
*Current contraceptive use*								
Male condom	24/78	30.8	1					
Other (hormonal, intrauterine device)	92/226	40.7	1.5	0.9-2.7	0.1			
								
*Visited gynecologist with in last 12 months*								
Yes	63/158	39.9	1					
No	57/168	33.9	0.8	0.5-1.2	0.3			
								
Any* cervical cancer screening with in last 12 months								
Yes	55/129	42.6	1					
No	65/197	33.0	0.7	0.4-1.0	0.08			

* Opportunistic or systematic cervical cancer screening

## Discussion

Cervical cancer is the second most common cancer in women worldwide. It is the sixth most frequent cancer among Estonian women of all ages, and the second most frequent cancer among young Estonian women (aged between 15 and 44 years) [17]. The availability of effective HPV vaccines has prompted a discussion about their use within public health vaccine programs. Geographical data on the HPV type distribution for women with cervical cancers as well as among women in the general population are essential for estimating the impact of cervical screening and vaccination programs [18]. Several recent studies have documented pre-vaccination HPV prevalence [4,19-23] varying according to the study design (sampling), study cohorts (clinic based, population based) and time period. These studies have typically used cervical samples obtained during a gynecological examination in the context of routine screening. In countries where cervical cancer screening is not widely implemented, such studies may not represent the general population and a non-invasive method acceptable to a representative sample of women is needed. One such option is the self-obtained vaginal sample. Several studies have documented that self-sampling has similar sensitivity to physician-obtained sampling to detect HPV DNA, suggesting that it is a viable screening option and may be a suitable alternative method for studies on HPV transmission and vaccine trials [24,25]. Studies utilizing HPV DNA testing of asymptomatic women in the general population estimate the prevalence of HPV infection to be in the range of 2-44% [26]. Self-collected vaginal samples likely pick up a mixture of cervical and vaginal cells. Previous analyses have established that genotypes within phylogenetic species tend to be similar with respect to the anatomic site of infection [27,28]. Studies comparing HPV detection in paired cervical and vaginal specimens have shown that the carcinogenic HPV types (alpha9 phylogenetic species, i.e. HPV 16) have a similar affinity for vaginal and cervical epithelium (resulting in similar recorded prevalences for any carcinogenic HPV type in vaginal and cervical specimens) [28], but non-carcinogenic HPV types of the alpha3/alpha15 (i.e. HPV 61) phylogenetic species may have a stronger affinity for vaginal epithelium [28].

Consistent with published data, we found that HPV 16 was the most prevalent HPV type (6.4%). We found a relatively low HPV 18 prevalence. In the light of studies documenting similar prevalence of any high-risk HPV in paired cervical and vaginal specimens this finding is of importance [28,29], and we would expect to see a low prevalence of HPV 18 in cervical samples from the same population. A low prevalence of HPV 18 among women from post-Soviet countries has been documented in other studies [30]. HPV 6 and 11 (alpha10 phylogenetic species) are relatively rarely isolated from vaginal/cervical specimens (HPV 6 < 1.5% and HPV 11 < 0.5% from cervical and vaginal specimens in Castle et al 2007 [29]; HPV 6 < 2% and HPV 11 < 0.5% from cervical specimens in Butsch Kovacic et al 2006 [31]; and HPV 6 from vaginal specimens either physician or patient-collected < 3.5% in Moscicki et al [32]), findings that are similar to those in our study.

Based on the most recent studies (Table 4) we surmise that Estonia represents a typical eastern European country with higher HPV and high-risk HPV prevalences than those reported from neighboring countries with well-developed population-based cervical cancer screening programs (i.e. Finland, Sweden, Denmark).

**Table 4:** Studies describing HPV and high-risk type-specific prevalence from different countries

Country	Study design	Test method	HPV prevalence	Comment	**Cervical cancer incidence rate per 100 000 **[17]^***a***^
Denmark Kjær SK, 2008 [20]	Population-based and opportunistic screening N = 11617 aged 15-93 years	Hybrid capture 2 testing	Any - 26.4% High risk - 23%	HPV peak prevalence at the age 20-24 (50.2%) Most prevalent high risk HPV 16 (6.0% of all women)	12.6

United Kingdom Sargent A, 2008 [23]	Routine screening program, N = 24510 aged 20-64 years	Hybrid capture 2 testing	High risk All ages - 10.6% 20-29 y - 27.3% 30-39 y - 10.3% 40-49 y - 4.2% 50-64 y - 2.5%	Most prevalent high risk HPV 16, 18, 31, 51, 52	8.3

Finland Leinonen M, 2008 [19]	Population based screening, N = 16895 aged 25-65 years	Hybrid capture 2 testing	High risk - 7.5%	High risk HPV peak prevalence at the age 25-29 (24.1%)	4.3

Netherlands Coupe VMH, 2008 [22]	RC population based screening trial N = 45362 Aged 18-65	GP5+/6+-PCR EIA	High risk - 5.6%	High risk peak prevalence at the age 18-24 (21.2%) Most prevalent high risk HPV 16, 31,18	7.3

Norway Skjeldestad FE 2008 [21]	N = 898 Aged 16-24 (healthy, non-pregnant, sexually experienced)	PCR	HPV 6,11,16,18 - 26% HPV 16, 18 - 20.8% HPV 6, 11 - 9.6%	HPV 16 prevalence 16.3% HPV 18 prevalence 7.3%	10.4

USA Dunne EF, 2007 [4]	Probability sampling, nationally representative sample (NHANES 2003-4) N = 1921 Aged 14-59	PCR	Any - 26.8% High risk - 15.2% Low risk - 17.8%	HPV peak prevalence at the age 20 to 24 (44.8%) Most prevalent high risk HPV 53, 52, 59 Most prevalent low risk HPV 62, 84, 89	7.7

Latvia Silins I, 2004 [35]	Healthy controls from population registry N = 239 aged 18-89 (cervical cancer risk factor study)	PCR	High risk - 8% (HPV 16 and 18 only)		12.9

Russia Aleksandrova YN, 2000 [36]	Attendants of gynecological clinics N = 309 reproductive age; lacking the clinical or morphological symptoms of HPV-related diseases	PCR	Any - 29%	HPV were overrepresented among women reporting excessive number of contraceptive abortions	11.9

Three NIS of former Soviet Union Kulmala SM, 2007 [30]	Consecutive out-patient clinic attendees N = 3187 (STI clinics n = 722, gynecological patients n = 761, cervical cancer screening n = 1,692)	Hybrid capture 2 testing	High risk - 31.2% * STI clinic - 40.8%, * gynecological patients - 30.9%, * screening - 27.2%	Most prevalent high risk HPV 16, 31, 33	NA

Russia Kulmala SM, 2007 [30]	Consecutive out-patient clinic attendees N = 1967	Hybrid capture 2 testing	High risk - 33.4%,	Most prevalent high risk HPV 16, 31,33	11.9

Belarus Kulmala SM, 2007 [30]	Consecutive out-patient clinic attendees N = 568	Hybrid capture 2 testing	High risk - 27.5%,	Most prevalent high risk HPV 16, 33, 31	13.1

Latvia Kulmala SM, 2007 [30]	Consecutive out-patient clinic attendees N = 442	Hybrid capture 2 testing	High risk - 26.2%	Most prevalent high risk HPV 16, 33, 39	12.9

^*a *^Age-standardized rate

The overall HPV prevalence in our study population was high with the highest overall HPV prevalence among those aged 21-25 years (and the highest prevalence of high-risk HPV occurring in the 18-20 year age group) and declining with age. It is well known that sexually active young adults are most at risk for acquiring HPV. Beside young age, predictors of HPV infection included younger age at first sexual intercourse and higher number of sexual partners in the 12 months before the study. Our results are also in line with existing information on HPV epidemiology [26,33,34].

Our study has several limitations. This study was not originally designed to assess HPV prevalence, thus we do not have data on cervical cytology from participating women. The degree to which the study is representative of the general population may be affected by the low response rate and selection factors associated with response. It is very probable that non-responders and women who were not reachable are at higher risk of exposure to HPV. However, in our study, the probability of not responding was not related to ethnicity, residency or age, despite the correlation between young age and infection. Also, we were unable to include preadolescent women as this would have been ethically unjustifiable and impractical from a logistical standpoint. While it is reasonable to assume that the prevalence estimates from this study are somewhat inflated due to selective participation, the estimate is probably better than that based on traditional clinic-based findings which are not representative in Estonia.

## Conclusion

Several factors affecting cervical cancer control are changing, including a better understanding of the natural history of HPV, reliable assays for detecting high-risk HPV infections, and the availability of effective preventive vaccines. However, there are important differences in the relevant policy questions for different settings. Local data including information on HPV (type specific) prevalence is needed for modeling in a decision analytic framework to identify those factors most likely to influence outcomes, provide insight into the cost-effectiveness of different strategies, and assist in early decision-making when weighed against equity, public preferences, and political and cultural constraints. Our study provides valuable baseline data about prevalence and type distribution of HPV prior to the introduction of HPV vaccination.

## Competing interests

The content of this paper has not been published elsewhere, nor is it being considered elsewhere, nor are there any conflicts of interest contained therein.

## Authors' contributions

AU, LAM and JD designed the study and outlined the analysis. MR performed the needed statistical analyses. LK was responsible for the laboratory work needed. AU wrote the first draft of the manuscript. All authors contributed to revising the manuscript and have approved the final manuscript.

## Pre-publication history

The pre-publication history for this paper can be accessed here:

http://www.biomedcentral.com/1471-2334/10/63/prepub

## References

[B1] Koutsky L Epidemiology of genital human papillomavirus infection Am J Med 1997 102 3 8 10.1016/S0002-9343(97)00177-0 9217656

[B2] Clifford GM Smith JS Aguardo T Franceschi S Comparison of HPV type distribution in high-grade cervical lesions and cervical cancer: a meta-analysis Br J Cancer 2003 89 101 105 10.1038/sj.bjc.6601024 12838308 PMC2394204

[B3] Anonymous European Medicines Agency, EPARs for authorised medicinal products for human use (http://www.emea.europa.eu/humandocs/Humans/EPAR/gardasil/gardasil.htm, http://www.emea.europa.eu/humandocs/Humans/EPAR/cervarix/cervarix.htm, accessed 29.09.2008)

[B4] Dunne EF Unger ER Sternberg M McQuillan G Swan DC Patel SS Markowitz LE Prevalence of HPV Infection Among Females in the United States JAMA 2007 297 8 813 819 10.1001/jama.297.8.813 17327523

[B5] Arbyn M Dillner J Review of current knowledge on HPV vaccination: an appendix to the European Guidelines for Quality Assurance in Cervical Cancer Screening J Clin Virol 2007 38 3 189 97 10.1016/j.jcv.2006.12.009 17258503

[B6] Haug CJ Human Papillomavirus Vaccination -- Reasons for Caution N Engl J Med 2008 359 861 862 10.1056/NEJMe0804638 18716305

[B7] Shah KV Daniel RW Tennant MK Shah N McKee JKT Rompalo A Diagnosis of human papillomavirus infection by dry vaginal swabs in military women Sex Transmit Infect 2001 77 260 264 10.1136/sti.77.4.260 PMC1744351 11463925

[B8] Brown DR Shew ML Qadadri B Neptune N Vargas M Tu W Juliar BE Breen TE Fortenberry JD A longitudinal study of genital human papillomavirus infection in a cohort of closely followed adolescent women J Infect Dis 2005 191 2 182 92 10.1086/426867 15609227 PMC2586143

[B9] Masek BJ Arora N Quinn N Aumakhan B Holden J Hardick A Agreda P Barnes M Gaydos CA Performance of three nucleic acid amplification tests for detection of Chlamydia trachomatis and Neisseria gonorrhoeae by use of self-collected vaginal swabs obtained via an Internet-based screening program J Clin Microbiol 2009 47 6 1663 7 10.1128/JCM.02387-08 19386838 PMC2691063

[B10] Gaydos CA Rompalo AM The Use of Urine and Self-obtained Vaginal Swabs for the Diagnosis of Sexually Transmitted Diseases Curr Infect Dis Rep 2002 4 2 148 157 10.1007/s11908-002-0057-4 11927048

[B11] Uusküla A Kals M Denks K Nurm U Kasesalu L Dehovitz J McNutt LA The prevalence of chlamydial infection in Estonia: a population-based survey Int J STD AIDS 2008 19 7 455 8 10.1258/ijsa.2008.007325 18574116 PMC2918246

[B12] Saiki RK Scharf S Faloona F Mullis KB Horn GT Erlich HA Arnheim N Enzymatic amplification of beta-globin genomic sequences and restriction site analysis for diagnosis of sickle cell anemia Science 1985 230 1350 4 10.1126/science.2999980 2999980

[B13] Gravitt PE Peyton CL Alessi TQ Wheeler CM Coutlée F Hildesheim A Schiffman MH Scott DR Apple RJ Improved Amplification of Genital Human Papillomaviruses J Clin Microbiol 2000 38 1 357 61 10618116 10.1128/jcm.38.1.357-361.2000 PMC88724

[B14] Meyer T Arndt R Stockfleth E Flammann HT Wolf H Reischl U Strategy for typing human papillomaviruses by RFLP analysis of PCR products and subsequent hybridization with a generic probe Biotechniques 1995 19 4 632 9 8777058

[B15] Muñoz N Castellsagué X de González AB Gissmann L Chapter 1: HPV in the etiology of human cancer Vaccine 2006 24 S3 S1 S10 10.1016/j.vaccine.2006.05.115 16949995

[B16] Victora CG Huttly SR Fuchs SC Olinto MT The role of conceptual frameworks in epidemiological analysis: a hierarchical approach International Journal of Epidemiology 1997 26 1 224 227 10.1093/ije/26.1.224 9126524

[B17] Anonymous Human Papillomavirus and Cervical Cancer. Summary Report 2007 WHO/ICO Information Centre on HPV and Cervical Cancer (HPV Information Centre) http://www.who.int/hpvcentre/statistics/dynamic/ico/country_pdf/XEX.pdf?CFID=1179971&CFTOKEN=54301985 accessed 10.09.2008

[B18] Goldie SJ Goldhaber-Fiebert JD Garnett GP Chapter 18: Public health policy for cervical cancer prevention: The role of decision science, economic evaluation, and mathematical modeling Vaccine 2006 24 Suppl 3 S155 63 10.1016/j.vaccine.2006.05.112 16950003

[B19] Leinonen M Kotaniemi-Talonen L Anttila A Dyba T Tarkkanen J Nieminen P Prevalence of oncogenic human papillomavirus infection in an organised screening population in Finland Int J Cancer 2008 123 6 1344 9 10.1002/ijc.23670 18566992

[B20] Kjær SK Breugelmans G Munk C Junge J Watson M Iftner T Population-based prevalence, type- and age-specific distribution of HPV in women before introduction of an HPV-vaccination program in Denmark Int J Cancer 2008 123 8 1864 1870 10.1002/ijc.23712 18661520

[B21] Skjeldestad FE Mehta V Sings HL Øvreness T Turpin J Su L Boerckel P Roberts C Bryan J Jansen KU Esser MT Liaw KL Seroprevalence and genital DNA prevalence of HPV types 6, 11, 16 and 18 in a cohort of young Norwegian women: study design and cohort characteristics Acta Obstet Gynecol Scand 2008 87 1 81 8 10.1080/00016340701714703 17943470

[B22] Coupe VMH Berkhof J Bulkmans NWJ Snijders PJF Meijer CJLM Age-dependent prevalence of 14 high-risk HPV types in the Netherlands: implications for prophylactic vaccination and screening Br J Cancer 2008 98 646 651 10.1038/sj.bjc.6604162 18182990 PMC2243156

[B23] Sargent A Bailey A Almonte M Turner A Thomson C Peto J Desai M Mather J Moss S Roberts C Kitchener HC ARTISTIC Study Group Prevalence of type-specific HPV infection by age and grade of cervical cytology: data from the ARTISTIC trial Br J Cancer 2008 98 10 1704 9 10.1038/sj.bjc.6604324 18392052 PMC2391119

[B24] Petignat P Faltin DL Bruchim I Tramèr MR Franco EL Coutlée F Are self-collected samples comparable to physician-collected cervical specimens for human papillomavirus DNA testing? A systematic review and meta-analysis Gynecol Oncol 2007 105 2 530 5 10.1016/j.ygyno.2007.01.023 17335880

[B25] Stewart DE Gagliardi A Johnston M Howlett R Barata P Lewis N Oliver T Mai V HPV Self-collection Guidelines Panel. Self-collected samples for testing of oncogenic human papillomavirus: a systematic review J Obstet Gynaecol Can 2007 29 10 817 28 17915065 10.1016/s1701-2163(16)32636-6

[B26] Trottier H Franco EL The epidemiology of genital human papillomavirus infection Vaccine 2006 24 suppl 1 S1 S15 16406226 10.1016/j.vaccine.2005.09.054

[B27] Kreimer AR Katki HA Schiffman M Wheeler CM Castle PE ASCUS-LSIL Triage Study Group Viral determinants of human papillomavirus persistence following loop electrical excision procedure treatment for cervical intraepithelial neoplasia grade 2 or 3 Cancer Epidemiol Biomarkers Prev 2007 16 1 11 6 10.1158/1055-9965.EPI-06-0710 17220326

[B28] Winer RL Hughes JP Feng Q O'Reilly S Kiviat NB Koutsky LA Comparison of Incident Cervical and Vulvar/Vaginal Human Papillomavirus Infections in Newly Sexually Active Young Women J Infect Dis 2009 199 6 815 8 10.1086/597118 19434913

[B29] Castle PE Rodriguez AC Porras C Herrero R Schiffman M Gonzalez P Hildesheim A Burk RD A comparison of cervical and vaginal human papillomavirus Sex Transm Dis 2007 34 11 849 55 10.1097/OLQ.0b013e318064c8c5 17621246 PMC3962831

[B30] Kulmala SM Shabalova IP Petrovitchev N Syrjänen KJ Gyllensten UB Syrjänen SM NIS Study Group Prevalence of the most common high-risk HPV genotypes among women in three new independent states of the former Soviet Union J Med Virol 2007 79 6 771 81 10.1002/jmv.20839 17457909

[B31] Butsch Kovacic M Castle PE Herrero R Schiffman M Sherman ME Wacholder S Rodriguez AC Hutchinson ML Bratti MC Hildesheim A Morales J Alfaro M Burk RD Relationships of human papillomavirus type, qualitative viral load, and age with cytologic abnormality Cancer Res 2006 66 20 10112 9 10.1158/0008-5472.CAN-06-1812 17047075

[B32] Moscicki AB Widdice L Ma Y Farhat S Miller-Benningfield S Jonte J Jay J Goodwin-Demedina C Hanson E Clayton L Shiboski S Comparison of natural histories of human papillomavirus (HPV) detected by clinician- and self-sampling Int J Cancer 2010 in press 20104517 10.1002/ijc.25199 PMC3132413

[B33] Shikary T Bernstein DI Jin Y Zimet GD Rosenthal SL Kahn JA Epidemiology and risk factors for human papillomavirus infection in a diverse sample of low-income young women J Clin Virol 2009 46 2 107 11 10.1016/j.jcv.2009.07.006 19665924 PMC2767195

[B34] Velicer C Zhu X Vuocolo S Liaw KL Saah A Prevalence and incidence of HPV genital infection in women Sex Transm Dis 2009 36 11 696 703 10.1097/OLQ.0b013e3181ad25ff 19652630

[B35] Silins I Wang X Tadesse A Jansen KU Schiller JT Avall-Lundqvist E Frankendal B Dillner J A population-based study of cervical carcinoma and HPV infection in Latvia Gynecol Oncol 2004 93 2 484 92 10.1016/j.ygyno.2004.01.044 15099967

[B36] Aleksandrova IN Lyshchëv AA Safronnikova NR Rudenko VI Imianitov EN Khnason KP Papillomavirus infection in healthy women from St. Petersburg Vopr Onkol 2000 46 2 175 9 10853416

